# A PROFILE OF CAREGIVING AMONG SEPSIS SURVIVORS RECEIVING POST-ACUTE HOME HEALTH CARE

**DOI:** 10.1093/geroni/igz038.799

**Published:** 2019-11-08

**Authors:** Jo-Ana D Chase, Christina R Whitehouse, Lizeyka Jordan, Kathryn H Bowles

**Affiliations:** 1 University of Missouri, Columbia, Missouri, United States; 2 Villanova University M. Louise Fitzpatrick College of Nursing, Villanova, Pennsylvania, United States; 3 Visiting Nurse Service of New York, New York, New York, United States; 4 University of Pennsylvania School of Nursing, Philadelphia, Pennsylvania, United States

## Abstract

Sepsis survivors transitioning from hospital-to-home are clinically complex. Family caregivers can face challenges managing patients’ care needs; however, skilled home health care (HHC) can serve as an important resource during this care transition. This study’s purpose was to describe caregiving needs among older sepsis survivors receiving post-acute HHC, and identify sources of unmet caregiving needs. We conducted a retrospective analysis of a national dataset of Medicare beneficiaries starting a new HHC episode who were after hospital discharge for sepsis between 2013 and 2014 (n=165,228). All patients received at least one HHC visit the first week after hospital discharge. Caregiving activities included seven items from the start of care Outcome and Assessment Information Set. Descriptive statistics were used to examine types of caregiving activities and needs, demographics, and clinical information. Proportions of patients with unmet caregiving needs ranged from 9%-29%, with the largest proportion of unmet needs in activities of daily living (ADL) assistance (29%), medication administration (28%), and medical procedures/treatments (25%). Unmet caregiving needs across activities were largely due to a caregiver needing training/supportive services (75%-88%), suggesting that many sepsis survivors receiving HHC have caregivers who are available to help, but who lack the knowledge and skills to manage patients’ complex care needs. Thus, HHC providers should address caregiving training and support needs, especially related to assistance with ADLs, medication administration, and medical procedures/treatments. Future research is needed to determine specific educational strategies for caregiver training and support, especially related to skills and knowledge assessment, and training delivery and monitoring.

